# Estimation of elevated intracranial pressure in infants with hydroce-phalus by using transcranial Doppler velocimetry with fontanel compression

**DOI:** 10.1038/s41598-018-30274-3

**Published:** 2018-08-07

**Authors:** Teiko Yoshizuka, Masahiro Kinoshita, Sachiko Iwata, Kennosuke Tsuda, Takenori Kato, Mamoru Saikusa, Ryota Shindou, Naoko Hara, Eimei Harada, Sachio Takashima, Nobuyuki Takeshige, Shinji Saitoh, Yushiro Yamashita, Osuke Iwata

**Affiliations:** 1 Department of Paediatrics and Child Health, Kurume University School of Medicine, Fukuoka, Japan; 2 Center for Human Development and Family Science, Department of Neonatology and Pediatrics, Nagoya City University Graduate School of Medical Sciences, Nagoya, Aichi Japan; 3 Yanagawa Institute for Developmental Disabilities, International University of Health and Welfare, Fukuoka, Japan; 4 Department of Neurosurgery, Kurume University School of Medicine, Fukuoka, Japan

## Abstract

For infants with acute progressive hydrocephalus, invasive drainage of cerebrospinal fluid (CSF) is performed until a ventriculo-peritoneal shunt can be inserted. Surrogate markers of intracranial pressure (ICP) may help optimise the timing of invasive procedures. To assess whether RI with/without fontanel compression helps distinguish between infants with normal (<5 cmH_2_O), mild (5–11 cmH_2_O), and moderate (>11 cmH_2_O) ICP elevation, 74 ICP measures before/after CSF removal and 148 related Doppler measures of the middle cerebral artery were assessed. Higher RI was associated with fontanel compression, elevated ICP, and their interaction (all p < 0.001). Without compression, differences in RI were observed between normal and moderate (p < 0.001) and between mild and moderate ICP elevation (p = 0.033). With compression, differences in RI were observed for all pairwise comparisons among normal, mild, and moderate ICP elevation (all p < 0.001). Without compression, areas under the receiver-operating characteristic curve for prediction of mild and moderate ICP elevation were 0.664 (95% confidence interval (CI), 0.538–0.791; p = 0.020) and 0.727 (95% CI, 0.582–0.872; p = 0.004), respectively, which improved to 0.806 (95% CI, 0.703–0.910; p < 0.001) and 0.814 (95% CI, 0.707–0.921; p < 0.001), respectively, with compression. RI with fontanel compression provides improved discrimination of infants with absent, mild, and moderate ICP elevation.

## Introduction

Ventricular dilation is a common but serious complication of ventricular haemorrhage in preterm infants^1,2^. Vassilyadi *et al*. reported that 51% of preterm infants born <26 weeks gestation developed grade III/IV intraventricular haemorrhage, approximately 40% of whom developed progressive ventricular dilation^3^. Hydrocephalus associated with congenital diseases, such as Chiari malformation and meningomyelocele, is also common^4–6^. To ameliorate cerebral injury secondary to increased intracranial pressure (ICP), pharmacological treatments using diuretics have been applied^7^. However, a large-scale trial of infants with post-haemorrhagic ventriculomegaly showed no clinical benefit of acetazolamide and furosemide^8^. For hydrocephalus refractory to pharmacological treatments, surgical drainage of the cerebrospinal fluid (CSF) is necessary^9^, however, many premature infants are too small for placement of permanent drainage devices. In such cases, transient CSF removal is repeated until the time at which surgical intervention can be safely performed^10^. Lumbar puncture is a minimally invasive technique for transient CSF removal; however, the amount of CSF that can be removed by this procedure is generally much smaller than the recommended quantity of 10–20 ml/kg^10,11^. Thus, for efficient CSF drainage, transient ventricular drainage via the anterior fontanelle is often preferred, despite the risks of infection, bleeding, CSF leakage, and brain tissue destruction^10^. The timing of CSF removal needs to be determined based on precise ICP estimation to balance its risk and benefit.

A range of techniques have been developed to estimate ICP levels non-invasively, including simple assessment of head circumference and fontanel tension, two-dimensional ultrasound sonography (to assess morphological changes in the ventricular system and optic nerve diameter), Doppler velocimetry with/without arterial pressure waveform, and visual-evoked potentials^10,12,13^. Transcranial Doppler velocimetry is most commonly performed for newborn infants, taking advantage of their good acoustic windows. Reduced diastolic flow velocity in the cranial artery is suggestive of increased ICP. However, a range of clinical conditions, including patent ductus arteriosus, hypovolaemia, and low blood carbon dioxide tension, similarly reduce the diastolic flow velocity of cerebral arteries, leaving the estimation of mild to moderate ICP elevation difficult^14,15^. To improve the diagnostic accuracy of transcranial Doppler velocimetry, Taylor and colleagues obtained the flow pattern of the anterior cerebral artery, while gentle compression was applied to the anterior fontanelle^16^. Subsequently, the resistance index (RI) obtained during fontanel compression better reflected invasively measured ICP^17,18^. Although promising, this technique needs further validation, because of the relatively small number of ICP measures used for the analysis (25 measures obtained from 12 infants before and after CSF removal). In addition, that study assessed the relationship between ICP and RI in a full dataset, using a linear regression analysis. However, given that 6 of the 12 infants had significant ICP elevations >15 cmH_2_O, the diagnostic utility of this technique for mild to moderate ICP elevation needs to be assessed.

The aim of the current study was to assess whether intracranial Doppler velocimetry with fontanel compression discriminates infants with mild and moderate elevation of ICP from their peers with normal ICP.

## Results

### Clinical backgrounds, Doppler indices and fluid removal

Of 6 infants, 5 infants had post-haemorrhagic hydrocephalus, while the remaining infant had hydrocephalus associated with type-II Chiari malformation and surgically repaired meningomyelocele (Supplemental Table 1). All the infants were refractory to pharmacological treatment, and CSF drainage was started because of acute-progressive ventricular dilation (>2 cm/week; 4 infants), seizures (1 infant), or bradycardia (1 infant). None of these infants were diagnosed with obstructive hydrocephalus, and lumbar puncture was initially preferred in the 5 infants with post-haemorrhagic hydrocephalus. However, because of failure to meet the initial goal of removing 5 ml/kg of CSF, all 5 infants underwent fontanel puncture later.

**Figure 1:**
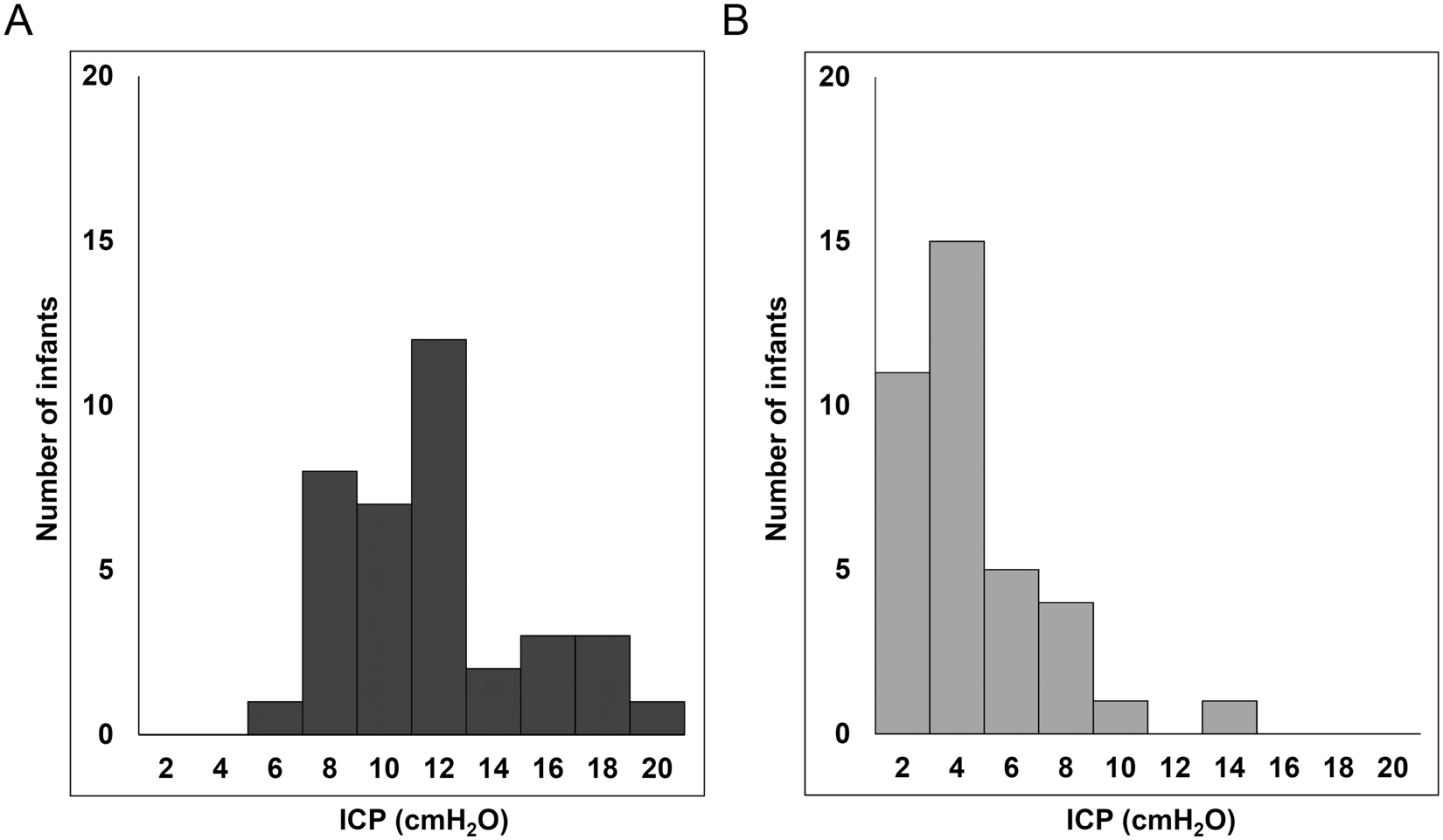
Distribution of intracranial pressure before (**A**) and after (**B**) fluid removal. Direct measurement of intracranial pressure (ICP) was performed 74 times before and after cerebrospinal fluid removal. Removal of cerebrospinal fluid resulted in an average reduction of ICP from 11.2 (3.4) cmH_2_O to 4.0 (2.7) cmH_2_O.

For the 6 infants, fontanel puncture was performed 37 times (1 to 12 times per infant) with a mean interval of 9.3 (6.1) days, and 74 ICP measurements were performed, at the beginning and end of each CSF removal. An average of 27.1 (14.1) ml of CSF was removed, resulting in an average reduction of ICP from 11.2 (3.4) cmH_2_O to 4.0 (2.7) cmH_2_O (Fig. 1). Major adverse events, such as infection, bleeding, apnoea, or persistent CSF leakage, were not recorded, except that one infant developed sinus bradycardia of 80 beats/min in association with the fontanel compression.

### Control variables of the Doppler indices

**Figure 2:**
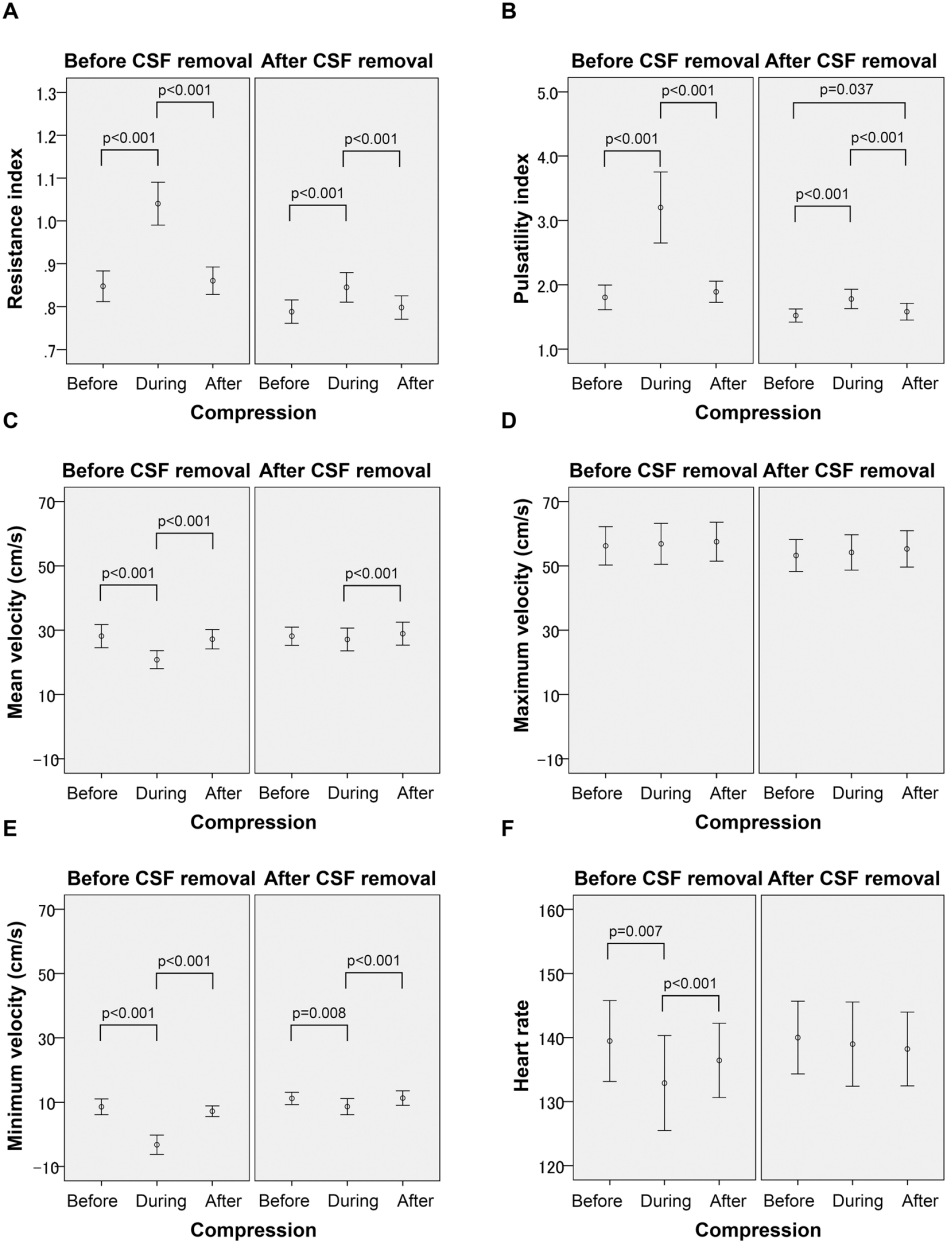
Temporal changes in Doppler indices with fontanel compression and cerebrospinal fluid removal. Fontanel compression was associated with increased resistance index and pulsatility index, and decreased minimum velocity, mean velocity, and heart rate, when adjusted for the removal of cerebrospinal fluid (CSF). CSF removal was associated with reduction of the resistance index, pulsatility index, and maximum velocity, and increases in minimum velocity and mean velocity (controlled for the fontanel compression). P-values indicated within the graph are taken from a simple effects test.

There were no significant differences in the Doppler indices before and after the fontanel compression (Doppler indices after compression are not considered further; Fig. 2). Gestational age was not associated with the Doppler indices (Table 1). Greater postnatal age was associated with the higher mean blood flow velocity (V_mean_; p = 0.011) and lower heart rate (p < 0.001). CSF removal was associated with reductions in RI (p < 0.001), pulsatility index (PI; p < 0.001), and the maximum blood flow velocity (V_max_; p = 0.001), and increases in the minimum flow velocity (V_min._; p < 0.001) and V_mean_ (p < 0.001). Fontanel compression was associated with increased RI (p < 0.001) and PI (p < 0.001), and decreased V_min_ (p < 0.001), V_mean_ (p < 0.001), and heart rate (p = 0.007). Compared with normal ICP, mild and moderate ICP elevation was associated with increased RI (both p < 0.001) and PI (both p < 0.001), and decreased V_min_ (p = 0.001 and p < 0.001, respectively). Significant interactions of fontanel compression with ICP levels were observed, leading to increased RI and PI, and decreased V_min_ (all p < 0.001; see Table 1 for interactions between fontanel compression and each ICP level).

**Table 1:** Dependence of Doppler indices on clinical backgrounds, fluid removal and fontanel compression.

Variables	Resistance index		Pulsatility index		Maximum velocity		Mean velocity		Minimum velocity		Heart rate	
	Β (95% CI)	P	Β (95% CI)	P	Β (95% CI)	P	Β (95% CI)	P	Β (95% CI)	P	Β (95% CI)	P
Gestational age^ab^ (week)	0.013 (−0.003, 0.029)	0.103	0.063 (−0.043, 0.170)	0.244	0.987 (−0.178, 2.151)	0.097	0.070 (−0.912, 1.053)	0.888	−0.572 (−1.432, 0.289)	0.193	1.391 (−1.170, 3.952)	0.287
Postnatal age^ab^ (day)	0.000 (−0.001, 0.001)	0.750	0.001 (−0.004, 0.006)	0.673	0.144 (0.017, 0.271)	0.026	0.061 (0.014, 0.107)	0.011	0.018 (−0.014, 0.050)	0.270	−0.219 (−0.339, −0.098)	<0.001
Timing to CSF removal^b^												
Before	Reference		Reference		Reference		Reference		Reference		Reference	
After	−0.127 (−0.138, −0.116)	<0.001	−0.852 (−1.084, −0.621)	<0.001	−2.824 (−4.551, −1.098)	0.001	3.147 (1.739, 4.555)	<0.001	7.237 (5.685, 8.788)	<0.001	3.311 (−6.570, −1.024)	0.007
Compression^a^												
No	Reference		Reference		Reference		Reference		Reference		Reference	
Yes	0.125 (0.102, 0.147)	<0.001	0.827 (0.626, 1.027)	<0.001	0.795 (−0.934, 2.523)	0.367	−4.177 (−5.544, −2.810)	<0.001	−7.170 (−8.625, −5.714)	<0.001	−3.797 (−6.570, −1.024)	0.007
ICP elevation^ab^												
Normal	Reference		Reference		Reference		Reference		Reference		Reference	
Mild	0.064 (0.032, 0.097)	<0.001	0.427 (0.199, 0.655)	<0.001	3.287 (0.228, 6.345)	0.035	−0.184 (−1.786, 1.417)	0.821	−3.365 (−5.261, −1.469)	0.001	3.577 (−0.966, 8.120)	0.123
Moderate	0.157 (0.123, 0.190)	<0.001	1.020 (0.581, 1.459)	<0.001	3.235 (−5.434, 11.904)	0.464	−3.657 (−8.488, 1.174)	0.138	−8.658 (−10.154, −7.162)	<0.001	4.843 (−1.786, 11.472)	0.152
Compression × ICP level^a^		<0.001		<0.001		0.953		<0.001		<0.001		0.241
x Normal	Reference		Reference		Not applicable^c^		Reference		Reference		Not applicable^c^	
x Mild	0.098 (0.043, 0.153)	0.001	0.597 (0.155, 1.04)	0.008			−3.16 (−5.966, −0.354)	0.027	3.577 (−0.966, 8.120)	0.123		
x Moderate	0.117 (0.056, 0.178)	<0.001	1.299 (0.684, 1.914)	<0.001			−6.03 (−9.478, −2.582)	0.001	−7.806 (−11.351, −4.26)	<0.001		

Dependence of Doppler indices on gestational age, postnatal age, cerebrospinal fluid (CSF) removal, fontanel compression and intracranial pressure (ICP) are presented as regression coefficient (B) and 95% confidence interval (CI). Findings are adjusted for CSF removal^a^ and fontanel compressionb.

**Figure 3:**
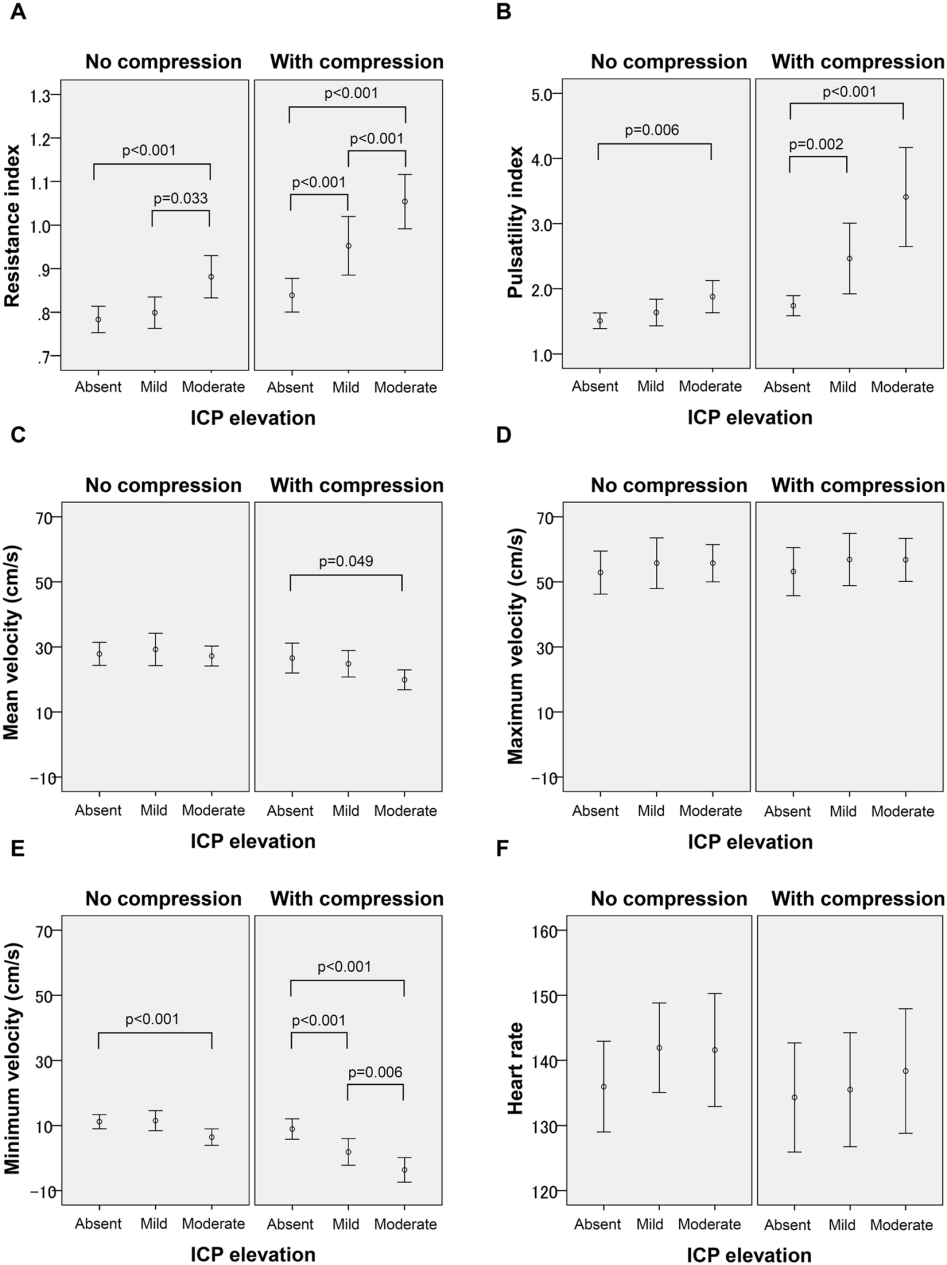
Fontanel compression, intracranial pressure, and Doppler indices. Resistance index (**A**) pulsatility index (**B**) mean velocity (**C**) maximum velocity (**D**) minimum velocity (**E**) and heart rate (**F**) with or without the fontanel compression are shown for three groups of no, mildly and moderately increased intracranial pressure (ICP). The difference in the resistance index, pulsatility index, and the minimum velocity among three ICP levels became more prominent with fontanel compression.

^c^Interactions between fontanel compression and ICP levels were not assessed when the main effect of these variables was not observed (see Fig. 3 for findings from pairwise comparisons using simple effects tests).

A simple effects test showed that RI without compression was higher with moderately elevated ICP than with normal (p < 0.001) and mildly (p = 0.033) elevated ICP. RI with compression differed for all pairwise comparisons among no, mildly, and moderately elevated ICP (all p < 0.001) (Fig. 3). PI without compression was higher with moderately elevated ICP than with normal ICP (p = 0.006). PI with compression was higher with moderately (p < 0.001) and mildly (p = 0.002) elevated ICP than with normal ICP. V_min_ without compression was lower with moderately elevated ICP than with normal ICP (p = 0.001); V_min_ with compression differed for all pairwise comparisons (p < 0.001 between normal and mild, and between normal and moderate ICP elevation; p = 0.006 between mild and moderate ICP elevation).

### Diagnostic value of Doppler indices for elevated intracranial pressure

**Figure 4:**
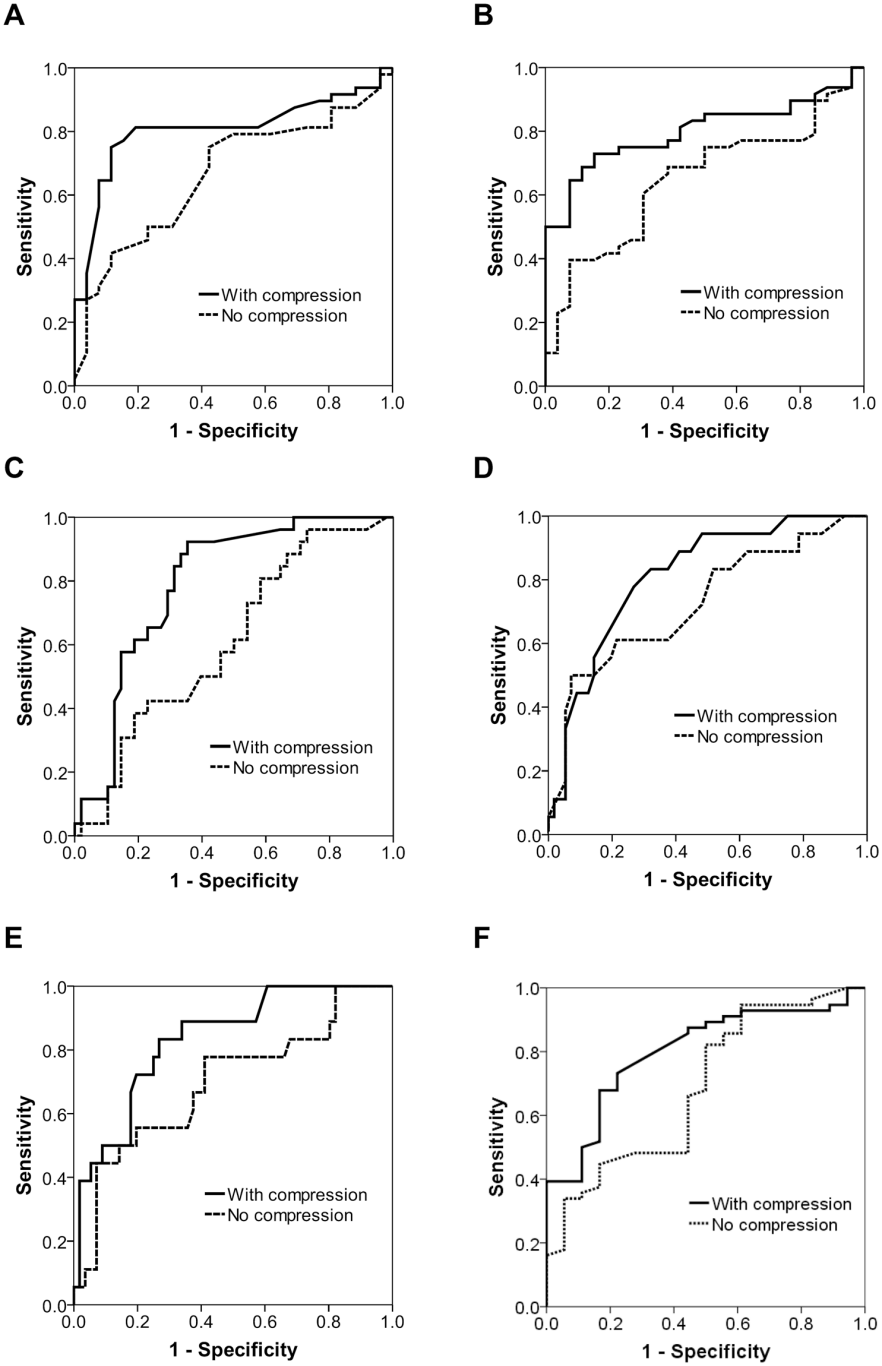
Receiver operating characteristic of Doppler indices for prediction of elevated intracranial pressure. Receiver operating characteristic of resistance index (**A** and **D**), pulsatility index (**B** and **E**) and minimum velocity (**C** and **F**) to predict mild (**A**–**C**) and moderate (**D**–**F**) elevation of intracranial pressure (ICP). The area under the curve values for prediction by the resistance index and pulsatility index of mild and moderate ICP elevation were improved by the fontanel compression.

A receiver-operating characteristic analysis showed that the area under the curve (AUC) values of RI for prediction of mild and moderate ICP elevation were 0.664 (95% CI, 0.538–0.791; p = 0.020) and 0.727 (95% CI, 0.582–0.872; p = 0.004), respectively (Fig. 4 and Supplemental Table 2), which were improved to 0.806 (95% CI, 0.703–0.910; p < 0.001) and 0.814 (95% CI, 0.707–0.921; p < 0.001), respectively, with fontanel compression. PI without compression predicted mild and moderate ICP elevation with AUCs of 0.648 (95% CI, 0.520–0.775; p = 0.037) and 0.695 (95% CI, 0.545–0.846; p = 0.013), respectively, which were improved to 0.800 (95% CI, 0.699–0.900; p < 0.001) and 0.833 (95% CI, 0.730–0.936; p < 0.001), respectively, with fontanel compression. The optimal cut-offs to predict mild and moderate ICP elevation were 0.87 (positive predictive value, 0.897; negative predictive value, 0.629) and 1.00 (positive predictive value, 0.438; negative predictive value, 0.911), respectively, for RI with compression, and 2.05 (positive predictive value, 0.897; negative predictive value, 0.629) and 2.08 (positive predictive value, 0.500; negative predictive value, 0.932), respectively, for PI with compression (Supplemental Table 2).

## Discussion

No non-invasive method has yet been established for distinguishing absent, mild and moderate ICP elevation in hydrocephalic infants, despite the clinical importance of this problem^19–22^. Building on the proposal by Taylor and colleagues^17^, we demonstrated that subtle ICP differences can be distinguished using RI and PI with fontanel compression. With further validation studies, Doppler velocimetry with fontanel compression might help optimise the indication and timing of both palliative and permanent drainage of CSF.

Doppler indices of cerebral arteries have been used as a simple tool for evaluating impaired cerebral perfusion in infants^14,15^. The mechanism by which elevated ICP induces an increase in RI and PI remains unknown, but it has been speculated that excessive ventricular dilation and subsequent elevation of ICP alter the diastolic flow velocity in cerebral arteries, leading to an increase in RI and PI. Whitelaw and Aquilina reported that RI values greater than 0.85 are suggestive of increased ICP, and RI greater than 1.0 indicates impaired perfusion in infants with post-haemorrhagic ventricular dilation, provided the ductus arteriosus is closed^10^. Although these Doppler indices can be repeated non-invasively at the bedside, their diagnostic accuracy is limited, because diastolic flow patterns depend on a range of intrinsic and extrinsic clinical conditions other than ICP, including blood gas contents, cardiac function, and the state of the ductus arteriosus^23,24^. In addition, the influence of mild to moderate ICP elevation to the diastolic flow pattern might be counteracted by compensatory regulation of vascular tone.

Consistent with the findings of Taylor and colleagues^17^, gentle compression of the anterior fontanelle increased the diagnostic value of RI and PI for elevated ICP. Our data highlights the value of fontanel compression for distinguishing mild and moderate increases in ICP from normal levels. Increases in both cranial volume and ICP might explain the changes in the flow pattern, but an important question remains concerning the mechanism of velocimetry changes with compression, because the increases in cranial volume would be similar between infants with and without ICP elevation. In a simple hemispheric model of the head with a circumference of 30 cm and an anterior fontanelle of 3 × 3 cm, the estimated increase in cranial volume associated with gentle compression of the anterior fontanelle is less than 1 ml, whereas the volume of the hemisphere is 228 ml. Considering the relatively high cerebrospinal compliance observed in our study cohort (4.0 ml/cmH_2_O in average), ICP changes caused by gentle fontanel compression is estimated to be <0.3 cmH_2_O. Nevertheless, a small but sudden increase in the cranial volume might be enough to decouple the balance between cerebral blood flow demand and the supply provided by maximal compensatory regulation. Interestingly, even for fontanel compression, Taylor and colleagues reported their impression that the response to compression might be decreased when it is repeatedly applied^17^. Further studies are required to understand the detailed mechanism by which flow patterns are altered in association with increased ICP and fontanel compression.

Our findings are based on a relatively large number of direct ICP measures and related Doppler velocimetry records. However, the study population was limited to 6 infants. To account for patient-specific velocimetry patterns and their responses to CSF removal and fontanel compression, the potential influence of repeated sampling was carefully controlled for by using a generalised estimating equation. Nevertheless, until future studies confirm the utility of this technique in larger study populations, our findings should be treated with caution. We confirmed that no significant ductus arteriosus shunt flow existed at the time of the study. However, other cardiac indices that might affect the flow pattern of cerebral arteries were not assessed. We grouped ICP into 3 levels according to the upper and lower quartiles of the binned ICP data, including values both before and after CSF removal. However, the actual ICP level at which CSF drainage is required might be different from these thresholds^22^. In the current study cohort, the mean ICP before CSF removal was 11.2 (3.4) cmH_2_O, which was associated with overt clinical signs of critical hydrocephalus without exception, suggesting the relevance of identifying ICP at around this level. In our study, fontanel compression caused no apparent adverse events except for brief sinus bradycardia at 80 beats/min. However, if fontanel compression uncouples the balance between blood flow demand and delivery, repetition of this manoeuvre might have deleterious effects. Taylor and colleagues monitored the blood flow pattern of the anterior cerebral artery^17^, but we used the left middle cerebral artery, because this allows compression of the anterior fontanelle to be performed while the Doppler velocimetry is continuously monitored. We also have an impression that the M1 segment of the middle cerebral artery provides relatively more stable velocimetry patterns (especially compared with the anterior cerebral artery) presumably because this segment of the middle cerebral artery forms a relatively long straight line pointing toward the transducer.

## Conclusions

Doppler velocimetry with fontanel compression provides improved discrimination of hydrocephalic infants with absent, mild and moderate-to-severe elevation of ICP. With further validation studies to confirm our findings, this technique might help optimise the indication and timing of both palliative and permanent CSF drainage, and improve safety and outcome for infants with hydrocephalus. Studies are also required to clarify the mechanism by which elevated ICP and transient fontanel compression alter the flow pattern of cerebral arteries.

## Materials and Methods

### Ethics approval and consent

This study was conducted in compliance with the Declaration of Helsinki and under the approval of the Ethics Committee of Kurume University School of Medicine. Written informed consent was obtained from a parent of each participating neonate.

### Study population

Between September 2015 and May 2016, 6 new-born infants (birth weight, 497–3074 g; gestational age, 23.0–37.9 weeks) were recruited who were hospitalised at a tertiary neonatal intensive care centre (Kurume University Hospital, Kurume, Fukuoka, Japan), developed hydrocephalus, and required transient CSF removal.

### Assessment and treatment of hydrocephalus

In this unit for extremely low birth weight infants and infants with ventricular dilation, B-mode cranial ultrasound is performed every day until day 5, and at least once a week thereafter, using a cardiovascular ultrasound sonography system (iE33 and 8–5 MHz Curved C8–5 micro-convex array transducer, Philips, Amsterdam, Netherlands). When ventriculomegaly is observed, head circumference is measured at least twice a week. Infants with acute progressive hydrocephalus are surgically provided with ventriculo-peritoneal shunts when the body weight of the infant reaches 2.5 kg. Until then, progressive ventriculomegaly greater than 1 cm/week is treated with parenteral furosemide (1–2 mg/kg/d) and/or asetazolamide (20–60 mg/kg/d). For acute progressive ventriculomegaly of greater than 2 cm/week, or for less severe ventriculomegaly with clinical symptoms, such as bradycardia, atypical apnoeic episodes, sunset eye sign, seizures, or poor oral feeding, lumbar puncture is initially performed, aiming to remove 5 ml/kg of CSF in the first application, and 10 ml/kg thereafter. When sufficient CSF cannot be removed via lumbar puncture, anterior fontanel puncture is performed by a neurosurgeon using an aseptic technique. During this procedure, gentle hand-holding is provided by a nurse, but analgesics and sedatives are not routinely given to infants. The skin is punctured at the lateral border of the anterior fontanelle, at least 2 cm lateral from the midline, advancing a 24-gauge needle directly towards the lateral ventricle. When CSF return is confirmed, the stylet is removed, and a syringe is connected via a short extension tube and a three-way cock. ICP is routinely recorded using a manometer tube connected to the three-way tap at the beginning and end of CSF removal.

### Data collection using Doppler velocimetry

Doppler studies were performed only before and after fontanel (but not lumbar) puncture because of uncertainties concerning the CSF removal and ICP measurement associated with lumbar puncture. To minimise the sampling bias, only three experienced neonatologists (M.K., K.T. and M.S.), who were unaware of the information obtained during CSF removal (e.g. ICP and the amount of CSF removed), performed the Doppler study. Identification numbers were assigned to each infant and study. Each study of an infant on a day of CSF drainage comprised 6 Doppler velocimetry waveforms, collected before and after the fontanel puncture. Shortly before the CSF removal, but before preparation for the aseptic procedure, the ultrasound probe was placed perpendicular to the left frontal temple of the infant, to visualise the M1 segment of the middle cerebral artery using the colour B-mode. The first Doppler velocimetry waveform was recorded when the infant was asleep or calmly awake for approximately 10 seconds, at 5 mm lateral from the proximal end of the M1 segment. Another physician then gently applied anterior fontanel compression until the surface became flat for 5–10 seconds. Fontanel compression was performed only once, to avoid tolerance of the vascular reactions to this manoeuvre^17^. Recording of the second velocimetry waveform was performed when the flow pattern became stable after the commencement of fontanel compression (typically within 5 seconds). The third recording was performed after the fontanel compression was terminated, and the flow pattern again became stable. Shortly after removing the drainage canula, this sequence was repeated to obtain the fourth (before compression), fifth (during compression) and sixth (after compression) velocimetry waveforms.

### Data analysis

Data analysis was performed based on the recorded velocimetry using the built-in software of the ultrasound system. V_max_, V_min_, V_mean_ and heart rate were calculated for at least 5 successive pulses using the automatic waveform tracing function of the built-in software. The automated velocimetry resulted were visually inspected for accuracy. RI and PI were also calculated using the following eqs (1 and 2).

1 $${\rm{RI}}=({{\rm{V}}}_{{\rm{\max }}}-{{\rm{V}}}_{{\rm{\min }}})/{{\rm{V}}}_{{\rm{\max }}}$$

2 $${\rm{PI}}=({{\rm{V}}}_{{\rm{\max }}}-{{\rm{V}}}_{{\rm{\min }}})/{{\rm{V}}}_{{\rm{mean}}}$$

The ICP measures were categorised into 3 levels according to the upper and lower quartiles of the full ICP measurement set: normal (<5 cmH_2_O), mild (5–11 cmH_2_O) elevation, and moderate (>11 cmH_2_O) elevation. For the folloing analyses, generalised estimating equations were used to properly deal with repeated sampling of the Doppler indices (i.e., V_max_, V_min_, V_mean._, RI, PI, and HR) from the same patient (on different days) and the same study (i.e., before and after CSF removal, and before, during and after fontanel compression) (SPSS ver. 20, IBM, Chicago, Illinoi, U.S.A.). First, the Doppler indices before and after (but not during) fontanel compression were compared. The results showed that the Doppler indices obtained before and after compression were statistically indistinguishable. For further analysis, only data obtained before and during (but not after) the fontanel compression were considered. Next, the dependence of the Doppler indices on gestational age at birth, postnatal age at the time of study, CSF removal (before vs. after CSF removal), fontanel compression (before and during compression), and ICP levels (normal, mild and moderate elevation) was assessed. The findings are presented incorporating the influence of CSF removal and/or fontanel compression. P values less than 0.013 were taken as significant (correcting for multiple comparisons over 4 independent variables). The interaction between the effects of fontanel compression and ICP levels on the Doppler indices was also assessed, followed by a post-hoc simple effects test. Finally, the predictive value of representative Doppler indices (i.e., RI and PI, with or without compression) for mild and moderate ICP elevation was assessed using the receiver operating characteristic curve.

## Electronic supplementary material

Online Supplementary Tables
