# Supplementary material for: Estimation of elevated intracranial pressure in infants with hydroce-phalus by using transcranial Doppler velocimetry with fontanel compression
Source: Sci Rep. 2018 Aug 7;8:11824. doi: 10.1038/s41598-018-30274-3 (PMC6081432; doi:10.1038/s41598-018-30274-3)
Supplement: Supplementary file 1 — Online Supplementary Tables [file 41598_2018_30274_MOESM1_ESM.docx]

**Online supplemental tables:**

**Title:**

Estimation of elevated intracranial pressure in infants with hydrocephalus by using transcranial Doppler velocimetry with fontanel compression

**Short title:**

Transcranial Doppler velocimetry with fontanel compression

**Authors:**

Teiko Yoshizuka^1^, Masahiro Kinoshita^1^, Sachiko Iwata^2^, Kennosuke Tsuda^2^, Takenori Kato^2^, Mamoru Saikusa^1^, Ryota Shindou^1^, Naoko Hara^1^, Eimei Harada^1^, Sachio Takashima^3^, Nobuyuki Takeshige^4^, Shinji Saitoh^2^, Yushiro Yamashita^1^, and Osuke Iwata^2^.

1. Department of Paediatrics and Child Health, Kurume University School of Medicine, Fukuoka, Japan.

2. Center for Human Development and Family Science, Department of Neonatology and Pediatrics, Nagoya City University Graduate School of Medical Sciences, Nagoya, Aichi, Japan.

3. Yanagawa Institute for Developmental Disabilities, International University of Health and Welfare, Fukuoka, Japan.

4. Department of Neurosurgery, Kurume University School of Medicine, Fukuoka, Japan.

**Correspondence to:**

Dr Osuke Iwata

Center for Human Development and Family Science, Department of Neonatology and Pediatrics, Nagoya City University Graduate School of Medical Sciences

1 Kawasumi, Mizuho, Nagoya, Aichi, 467-8602 Japan

E-mail: o.iwata@med.nagoya-cu.ac.jp

Tel: +81 52 851-5511

**Online supplemental table 1:** Clinical characteristics of the study population

| Patient identification number | Gestational age at birth (week) | Birth weight (kg) | Sex | Clinical course | Age at first CSF removal (day) | Number of CSF removal | Head circumference at | | | Age at VP-shunt insertion (day) |
| --- | --- | --- | --- | --- | --- | --- | --- | --- | --- | --- |
|  |  |  |  |  |  |  | Birth (cm) | First CSF removal (cm) | VP-shunt insertion (cm) |  |
| 1 | 23.0 | 0.72 | Male | Post-haemorrhagic hydrocephalus after grade II IVH on day 1 | 32 | 9 | 22.7 | 26.0 | 40.6 | 112 |
| 2 | 26.0 | 0.84 | Male | Post-haemorrhagic hydrocephalus after grade III IVH on day 2 | 26 | 6 | 24.8 | 28.5 | 45.2 | 95 |
| 3 | 26.0 | 0.77 | Male | Post-haemorrhagic hydrocephalus after grade III IVH on day 2 | 26 | 6 | 24.3 | 31.3 | 37.2 | 104 |
| 4 | 23.7 | 0.50 | Female | Post-haemorrhagic hydrocephalus after grade III IVH on day 2 | 38 | 12 | 19.5 | 23.8 | 37.6 | 163 |
| 5 | 23.9 | 0.68 | Female | Post-haemorrhagic hydrocephalus after grade IV IVH on day 2 | 36 | 4 | 22.5 | 25.2 | 44.0 | 120 |
| 6 | 37.9 | 3.07 | Male | Hydrocephalus associated with Chiari malformation type II | 22 | 4 | 41.0 | 44.0 | 41.5 | 81 |

Abbreviations: IVH, intraventricular haemorrhage. CSF, cerebrospinal fluid. VP, ventriculo-peritoneal.

**Online supplemental table 2:** Diagnostic properties of representative Doppler indices

|  | Compression | AUC | 95% CI | Cut-off | Sensitivity | Specificity | PPV | NPV | PLR | NLR |
| --- | --- | --- | --- | --- | --- | --- | --- | --- | --- | --- |
| ICP >5 cmH2O |  |  |  |  |  |  |  |  |  |  |
| Resistance index | No | 0.664 | 0.538 – 0.791 | 0.79 | 0.750 | 0.577 | 0.766 | 0.556 | 1.773 | 0.433 |
|  | Yes | 0.806 | 0.703 – 0.910 | 0.87 | 0.729 | 0.846 | 0.897 | 0.629 | 4.740 | 0.320 |
| Pulsatility index | No | 0.648 | 0.520 – 0.775 | 1.50 | 0.688 | 0.615 | 0.767 | 0.516 | 1.788 | 0.508 |
|  | Yes | 0.800 | 0.699 – 0.900 | 2.05 | 0.729 | 0.846 | 0.897 | 0.629 | 4.740 | 0.320 |
| Minimum velocity | No | 0.609 | 0.477 – 0.741 | 7.94 | 0.458 | 0.731 | 0.766 | 0.556 | 1.773 | 0.433 |
|  | Yes | 0.792 | 0.688 – 0.895 | 3.29 | 0.667 | 0.846 | 0.889 | 0.579 | 4.333 | 0.394 |
|  |  |  |  |  |  |  |  |  |  |  |
| ICP >11 cmH2O | |  |  |  |  |  |  |  |  |  |
| Resistance index | No | 0.727 | 0.582 – 0.872 | 0.85 | 0.611 | 0.786 | 0.478 | 0.863 | 2.852 | 0.495 |
|  | Yes | 0.814 | 0.707 – 0.921 | 1.00 | 0.778 | 0.732 | 0.483 | 0.911 | 2.904 | 0.304 |
| Pulsatility index | No | 0.695 | 0.545 – 0.846 | 1.54 | 0.778 | 0.589 | 0.378 | 0.892 | 1.894 | 0.377 |
|  | Yes | 0.833 | 0.730 – 0.936 | 2.28 | 0.833 | 0.732 | 0.500 | 0.932 | 3.111 | 0.228 |
| Minimum velocity | No | 0.686 | 0.541 – 0.831 | 5.60 | 0.500 | 0.821 | 0.444 | 0.821 | 2.489 | 0.676 |
|  | Yes | 0.801 | 0.691 – 0.911 | 0.00 | 0.778 | 0.732 | 0.483 | 0.911 | 2.904 | 0.304 |

Abbreviations: AUC, area under the curve. CI, confidence interval. ICP, intracranial pressure. NLR, negative likelihood ratio. NPV, negative predictive value. PLR, positive likelihood ratio. PPV, positive predictive value.
